# Clinical importance of the absolute count of neutrophils, lymphocytes, monocytes, and platelets in newly diagnosed hepatocellular carcinoma

**DOI:** 10.1038/s41598-021-82177-5

**Published:** 2021-01-28

**Authors:** Jeong Il Yu, Hee Chul Park, Gyu Sang Yoo, Changhoon Choi, Moon Seok Choi, Heerim Nam, Sun-Young Baek, Minsu Park

**Affiliations:** 1 Departments of Radiation Oncology, Samsung Medical Center, Sungkyunkwan University School of Medicine, 81 Irwon-ro, Gangnam-gu, Seoul, 06351 Republic of Korea; 2 Department of Medical Device Management and Research, Samsung Advanced Institute for Health Sciences and Technology, Sungkyunkwan University, Seoul, Republic of Korea; 3 Departments of Medicine, Samsung Medical Center, Sungkyunkwan University School of Medicine, Seoul, Republic of Korea; 4 Department of Radiation Oncology, Gangbook Samsung Hospital, Sungkyunkwan University School of Medicine, Seoul, Republic of Korea; 5 Statistics and Data Center, Research Institute for Future Medicine, Samsung Medical Center, Seoul, Republic of Korea; 6 Department of Statistics, Keimyung University, Daegu, Republic of Korea

**Keywords:** Cancer, Biomarkers, Oncology

## Abstract

Although several studies have confirmed the clinical significance of the systemic inflammation markers in hepatocellular carcinoma (HCC), evaluating the clinical significance of each blood cell remains to be conducted. We aimed to evaluate the clinical importance of absolute counts of blood cells in the overall survival (OS) of patients with newly diagnosed HCC. We recruited patient cohorts from the prospective registry of newly diagnosed and previously untreated HCC at Samsung Medical Center, which included a training set of 6619 patients (2005–2013) and a validation set of 2084 patients (2014–2016). More than three-quarters of all patients had hepatitis B virus (HBV)-related HCC in both training and validation sets. The optimal cutoff values of the absolute counts of neutrophils, lymphocytes, monocytes, and platelets were 3917, 488, 1379, and 22,100, respectively, which correlated significantly with OS. The absolute blood cell counts categorized by each optimal cutoff value significantly correlated with liver function status determined by Child–Pugh class/albumin-bilirubin (ALBI) grade and the HCC burden determined by several staging systems/portal vein tumor thrombosis. Although the prognostic model based on these blood cells (ABC model) showed a lower prognostic ability than the Japan Integrated Staging or ALBI-T staging systems, it provided significant discrimination of survival in the subgroups of ALBI-T and showed the highest prognostic ability in the present study in the training and validation sets. Absolute counts of blood cells are independently associated with OS, though it is also significantly associated with liver function and tumor burden in newly diagnosed HCC.

## Introduction

Despite the advances in the management of hepatocellular carcinoma (HCC), including an improved understanding of tumor biology, management of background liver disease, and recent achievement in the immuno-oncology field, HCC remains one of the leading causes of malignancy-related deaths worldwide^1^. Moreover, it is well known that the prognosis of HCC is mainly determined by the baseline liver function as well as the tumor burden^2–4^.

Recent studies have highlighted the crucial role of tumor microenvironment in the tumor progression and treatment response^5^. Furthermore, reports suggest subcategorization of white blood cells or platelets and their functions associated with tumor progression or suppression^6–8^. Numerous studies have confirmed the clinical significance of the neutrophil-to-lymphocyte ratio (NLR), lymphocyte-to-monocyte ratio (LMR), and platelet-to-lymphocyte ratio (PLR) in HCC^9–12^. Additionally, our group has also reported the prognostic significance of NLR, LMR, and PLR in newly diagnosed previously untreated patients with HCC using a large single institutional cohort data and validated the results in the same cohort registered at different times^13^.

Furthermore, the prognostic significance and/or actual role of each blood cell in various tumors and/or the clinical significance of the absolute blood cell count itself have been investigated^6–8,14^. The analysis from these studies considered absolute blood cell counts rather than the ratio of these cells to understand the mechanism of how each cell type regulates progression and/or suppression in tumor microenvironment. Moreover, these aspects have been actively studied and partially proved in several cancer types^6,8,15^. However, a detailed study evaluating the clinical significance of each blood cell in patients with HCC remains to be conducted.

Therefore, we investigated the clinical significance of the absolute counts of neutrophils, lymphocytes, monocytes, and platelets in newly diagnosed previously untreated patients with HCC.

## Results

### Definition and prognostic significance of optimal cutoff of absolute blood counts

**Figure 1:**
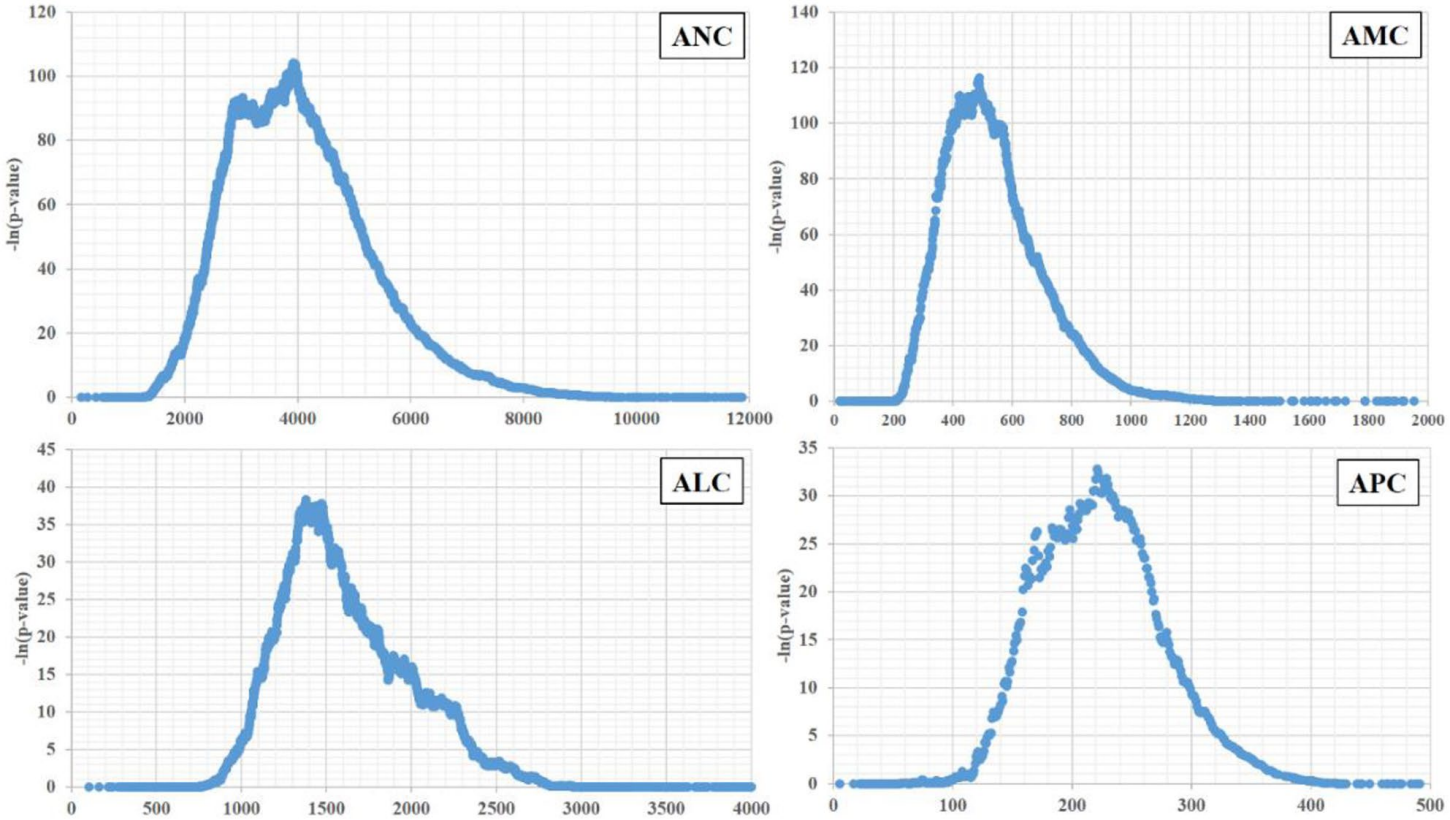
The graphs have been represented as − ln(p-value) of overall survival based on the specific count of blood cells: the cutoff values of 3917 in absolute neutrophil count, 488 in absolute monocyte count, 1379 in absolute lymphocyte count, and 22,100 in absolute platelet count created the greatest value of − ln(p-value).

**Figure 2:**
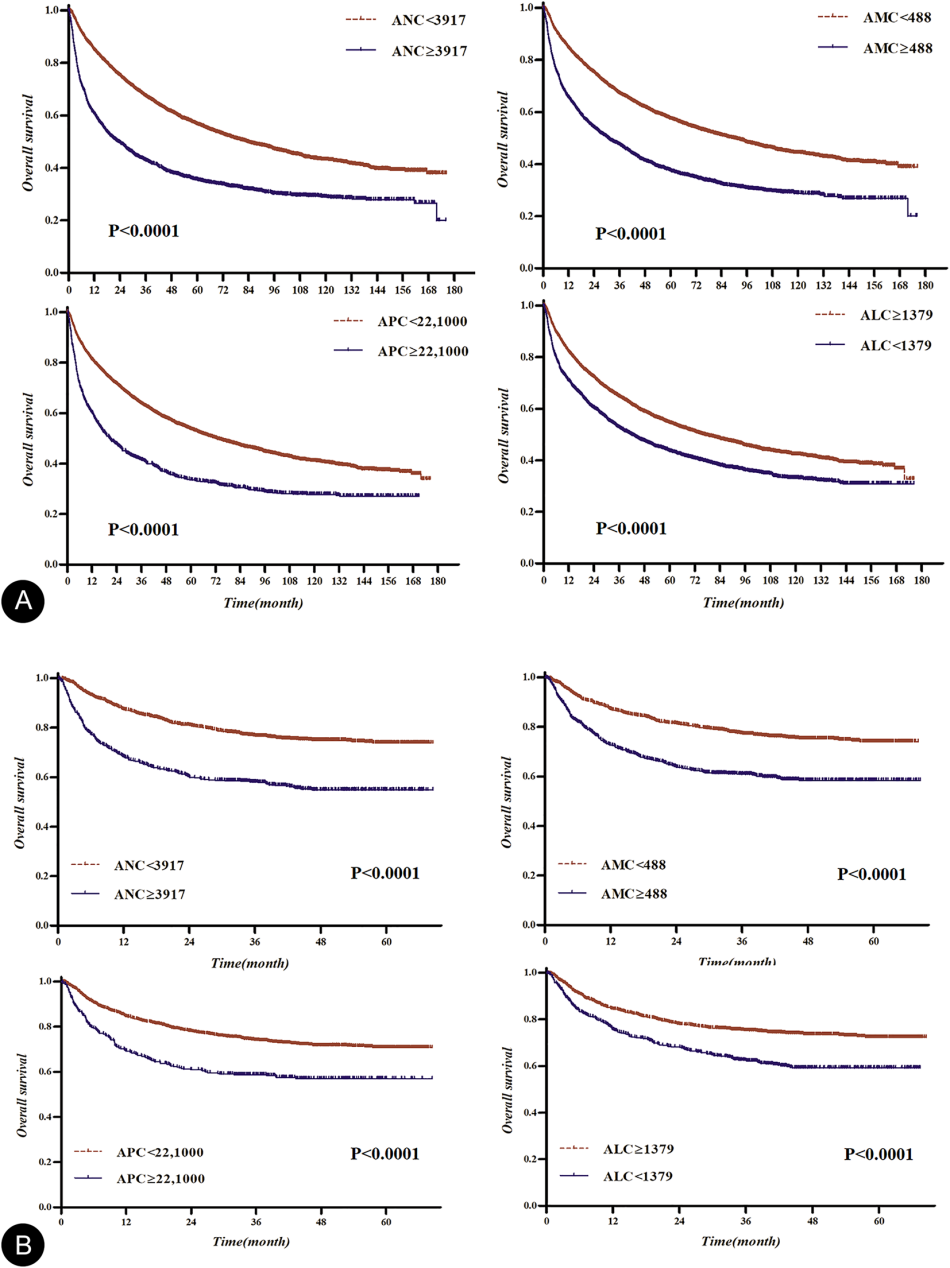
Kaplan–Meier curves for overall survival (OS) based on the optimal cutoff of absolute neutrophil, monocyte, lymphocyte, and platelet counts in the training and validation sets: the OS curves could be clearly divided according to the optimal cutoff counts in the training (**A**) and validation (**B**) sets.

The statistical significance of OS based on absolute count of each blood cell type was obtained (Supplemental Table 1), and the graphs were represented as − ln (*p*-value) for each absolute count (Fig. 1). The cutoff values for absolute neutrophil count (ANC), absolute monocyte count (AMC), absolute lymphocyte count (ALC), and absolute platelet count (APC) which determined by the point in time when the difference of OS was maximized using the log-rank test statistic were found to be 3917, 488, 1379, and 22,100, respectively. The Kaplan–Meier OS curves, based on the groups divided by the cutoff values identified in training and validation sets, are shown in Fig. 2. Moreover, our analysis suggests that ANC, AMC, and APC above their cutoffs is correlated with low OS, whereas ALC above cutoff is associated with high OS.

### Correlation between absolute blood counts and prognostic factors in HCC

The outcome of correlation between clinical variables, which were previously established prognostic factors, and blood cells as binary variable obtained by each optimal cutoff values has been summarized in Table 1 and Supplementary Table 2. Our analysis suggests significant correlation between most of the clinical variables and blood cells. Moreover, staging systems of HCC and/or portal vein tumor thrombosis (PVTT) and the initial treatment policies were significantly associated with absolute counts of all blood cells. Furthermore, the Eastern Cooperative Oncology Group (ECOG) performance status, Child–Pugh class, and ALBI grade were found to be significantly more correlated with AMC and ALC than with ANC and APC.

**Table 1:** The correlation between previously established prognostic variables and blood cells as a binary variable obtained using each optimal cutoff value.

Variables	ANC p-value	AMC p-value	ALC p-value	APC p-value
Age (years)	< 0.0001	0.322	0.926	< 0.0001
AFP (ng/mL)	< 0.0001	< 0.0001	< 0.0001	< 0.0001
PIVKA-II (mAU/mL)	< 0.0001	< 0.0001	0.65	< 0.0001
Body mass index	0.0008	0.627	< 0.0001	< 0.0001
Sex	< 0.0001	< 0.0001	< 0.0001	0.001
ECOG performance status	0.06	< 0.0001	< 0.0001	0.001
Cause of hepatitis	0.001	0.536	0.531	0.09
Child–Pugh Class	0.001	< 0.0001	< 0.0001	0.001
BCLC stage	< 0.0001	< 0.0001	< 0.0001	< 0.0001
ALBI grade	0.06	< 0.0001	< 0.0001	< 0.0001
Portal vein invasion	< 0.0001	< 0.0001	< 0.0001	< 0.0001
T stage	< 0.0001	< 0.0001	< 0.0001	< 0.0001
N stage	< 0.0001	< 0.0001	0.008	< 0.0001
M stage	< 0.0001	< 0.0001	< 0.0001	< 0.0001
Primary treatment	< 0.0001	< 0.0001	< 0.0001	< 0.0001

### Prognostic model based on absolute blood counts

**Figure 3:**
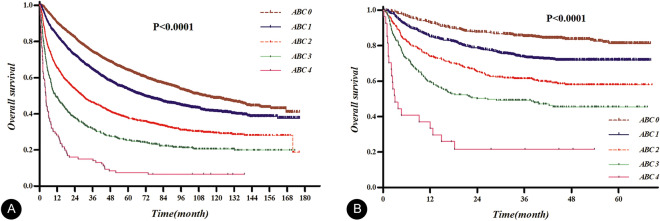
Kaplan–Meier curves for overall survival based on the ABC prognostic model established with absolute neutrophil count, absolute monocyte count, absolute lymphocyte count, and absolute platelet count: the curves were well separated based on the score of ABC in the training (**A**) and validation (**B**) sets.

Using the absolute blood counts as binary variable obtained by each optimal cutoff value, a new prognostic ABC model was established based on the number of risk factors observed above the cutoff values of ANC, AMC, and APC and below those for ALC. The Kaplan–Meier OS curves for the entire cohort of training and validation sets, based on the ABC model, are presented in Fig. 3. The difference in OS curves according to the ABC model was more prominent in the patients had HBV related HCC than in other patients, although there were statistical significant difference in both groups and both sets (all p < 0.001, Supplementary Fig. 1).

The OS was compared in the ABC model between the subgroups of training set classified according to the baseline liver function categorized based on Child–Pugh class. Based on our new model, the OS was found to be significantly correlated independent of the baseline liver function status, although the differences tended to decrease in the subgroup with Child–Pugh class C. Moreover, similar results were obtained in the validation set. Further application of the ABC model to subgroups in the training set allowed categorization of the modified International Union for Cancer Control (UICC) stage, where significant differences were observed in OS for all stages, except for stage I, and the validation set confirmed these results. The Kaplan–Meier OS curves of subgroups categorized with Child–Pugh class or UICC stage based on the new ABC model using training and validation sets are presented in Supplemental Fig. 2.

### Comparison of the prognostic performance of ALBI, Japan Integrated Staging Score (JIS), ALBI-T, and new ABC model

The distribution of patients and survival rates obtained in the training and validation sets based on each prognostic model are shown in Table 2. All prognostic models showed clear distinctions in the training and validation sets, and the ALBI-T indicated the best prognostic ability with highest C-index (0.702 in training set [95% confidence interval [CI] 0.712–0.716], 0.782 in validation set) and integrated area under curve (iAUC, 0.692 in training set [95% CI 0.684–0.699], 0.765 in validation set) values. Moreover, while the new ABC model well discriminated the prognoses in both the sets (C-index 0.626 in training [95% CI 0.617–0.636], and 0.652 in validation sets; iAUC 0.607 in training [95% CI 0.599–0.616], and 0.641 in validation sets), it showed relatively poor performance than the other models, including JIS and ALBI-T, especially in the group that presented with the best survival outcome. The Kaplan–Meier OS curves based on the prognostic models using the training and validation sets are presented in Supplemental Fig. 3.

**Table 2:** Patient distribution and survival rates based on each prognostic model.

Cohort	Prognostic model	Score	No (%)	Median survival (months)	Overall survival (%)			*p *value
					1 year	3 years	5 years	
Training	ALBI	1	3509 (53.0)	112.8	84.9	69.2	60.0	< 0.001
		2	2787 (42.1)	38.7	71.4	51.3	40.5	
		3	323 (4.9)	24.2	60.4	42.1	35.3	
	JIS	0	1044 (15.8)	NR	98.6	91.2	81.4	< 0.001
		1	2580 (39.0)	123.8	91.5	75.9	64.9	
		2	1718 (26.0)	35.6	75.4	49.8	38.0	
		3	1007 (15.2)	8.6	42.8	19.9	14.3	
		4	237 (3.6)	3.3	18.2	11.4	9.3	
		5	33 (0.5)	1.8	15.2	9.1	9.1	
	ALBI_T	0	656 (9.9)	NR	99.1	95.1	87.3	< 0.001
		1	2085 (31.5)	167.2	94.1	80.5	70.4	
		2	1966 (29.7)	57.7	83.5	61.5	48.9	
		3	1279 (19.3)	16.4	57.3	31.2	22.1	
		4	575 (8.7)	4.6	29.4	13.9	10.3	
		5	58 (0.9)	3.1	17.2	6.9	5.2	
	ABC	0	2103 (31.8)	112.3	90.2	74.2	64.0	< 0.001
		1	2604 (39.3)	70.9	83.1	64.4	53.4	
		2	1162 (17.6)	29.3	65.4	46.3	37.8	
		3	643 (9.7)	10.8	49.0	31.6	25.4	
		4	106 (1.6)	4.3	28.3	15.1	7.5	
Validation	ALBI	1	1503 (72.1)	NR	88.5	79.3	76.4	< 0.001
		2	537 (25.8)	38.3	65.4	50.4	47.2	
		3	44 (2.1)	8.8	47.7	45.2	40.2	
	JIS	0	333 (16.0)	NR	98.5	95.7	94.6	< 0.001
		1	809 (38.8)	NR	94.4	86.0	82.3	
		2	522 (25.0)	NR	80.7	66.0	62.6	
		3	331 (15.9)	13.0	51.4	33.6	31.0	
		4	79 (3.8)	3.1	24.1	15.4	13.2	
		5	10 (0.5)	2.2	–	–	–	
	ALBI_T	0	277 (13.3)	NR	98.6	97.1	96.4	< 0.001
		1	750 (36.0)	NR	95.6	88.1	84.5	
		2	532 (25.5)	NR	85.2	71.5	68.4	
		3	348 (16.7)	18.6	59.5	39.9	36.3	
		4	163 (7.8)	4.9	31.3	20.0	18.1	
		5	14 (0.7)	4.4	14.3	7.1	–	
	ABC	0	595 (28.6)	NR	92.9	85.3	81.2	< 0.001
		1	831 (39.9)	NR	85.1	73.6	71.9	
		2	393 (18.9)	NR	74.0	61.5	58.0	
		3	238 (11.4)	26.7	59.7	49.2	45.5	
		4	27 (1.3)	3.0	33.3	21.6	–	

*UICC* The Union for International Cancer Control, *JIS* Japanese Integrated Score, *ABC* absolute blood count based prognostic model, *NR* not reached.

### Prognostic significance of the ABC model in the subgroups classified with ALBI-T

**Figure 4:**
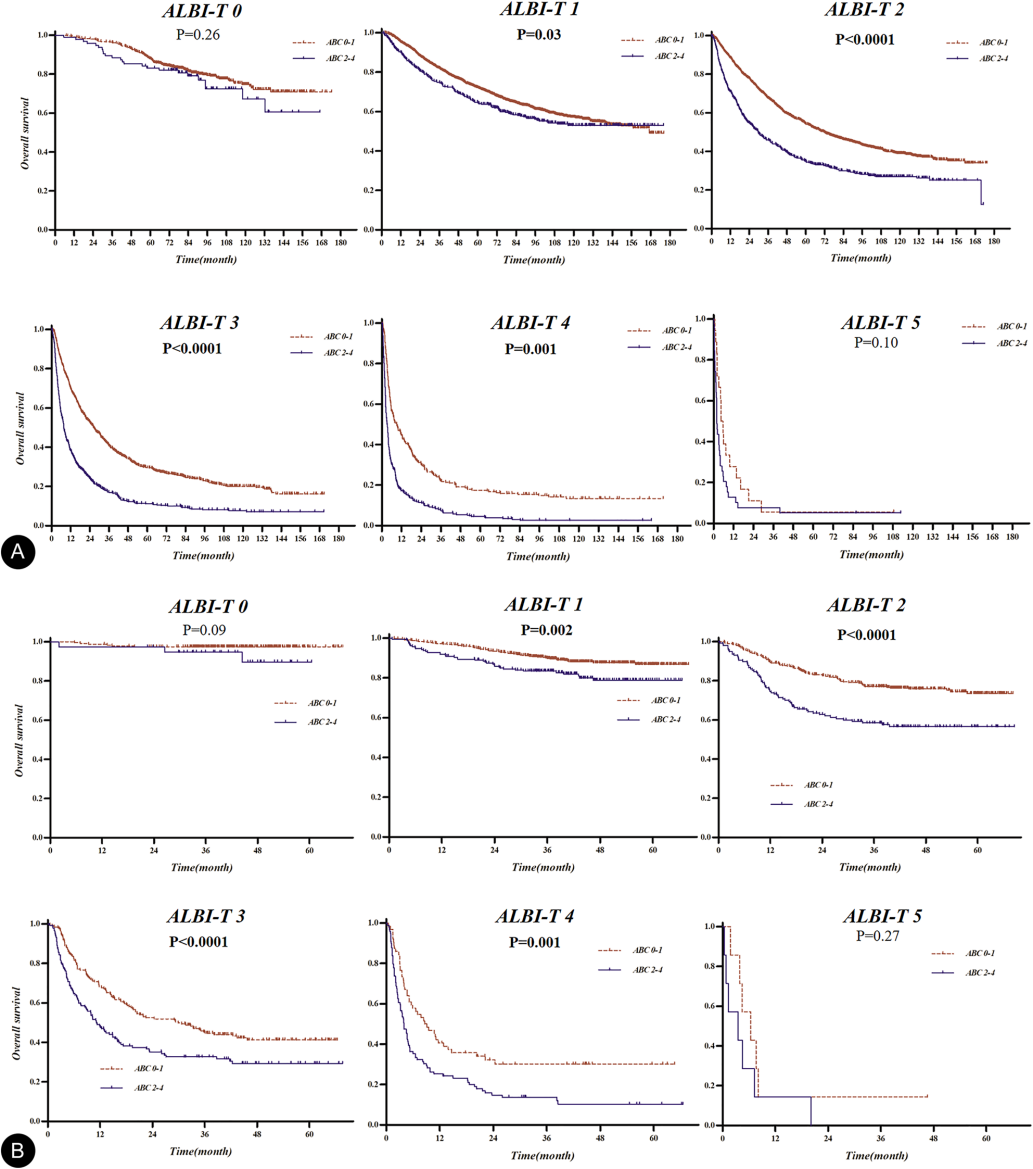
Kaplan–Meier curves for overall survival in the subgroups classified using albumin-bilirubin (ALBI)-T: the survival curves of subgroups were well separated based on the risk groups classified using the ABC model in both training (**A**) and validation (**B**) sets, except the ALBI-T 0 and 5 subgroups.

In the subgroups classified with ALBI-T, the ABC model showed significant prognostic differences in some subgroups in the training and validation sets (Supplemental Fig. 3). The ABC model categorizing into low-risk (0–1 point) and high-risk (2–4 point) could significantly distinguish the OS of subgroups identified by the ALBI-T in both the sets, except the ALBI-T “0” and “5” subgroups that showed best and worst prognoses, respectively (Fig. 4).

## Discussion

The absolute counts of blood cells, including neutrophils, monocytes, lymphocytes, and platelets, were significant prognostic factors and were significantly correlated with previously recognized prognostic factors, including baseline liver function status (Child–Pugh class or ALBI grade) and tumor burden (Barcelona Clinic Liver Cancer [BCLC]/UICC, stage or PVTT) in the present study conducted with the cohort of patients with newly diagnosed HCC.

The causality of chronic inflammation induced by viral infection, alcohol abuse, and/or steatosis in carcinogenesis of HCC is well known^16^. Especially, there is a growing evidence that formation of neutrophil extracellular trap (NET), one of the main antimicrobial functions of neutrophil, is an inflammatory microenvironment promoting cancer growth and proliferation^17^. And, recent experimental study shows that NET developed in the circumstance with abundant neutrophils, lead to replacement of the endogenous liver Kupffer cells by a population of infiltrating macrophages derived from monocytes^18^. Further, they could produce several inflammatory cytokines including interleukin (IL)-1β, IL-6, tumor necrosis factor (TNF)-α, and reactive oxygen species (ROS)^19,20^, which lead to an amplification of the inflammation, and result in steato-hepatitis. Moreover, the platelets have been found to initiate this process by inducing a rapid neutrophil activation and NET formation^21^. In fact, it is true that the process of steato-hepatitis is multifactorial, much more complicated and requires a lot of further research, the neutrophil, monocyte, and platelet are some of the crucial factors in this process^22^.

Next, the lymphocytes have been shown to play a fundamental role in immune mechanism against infectious microorganisms and various tumors. Reduction of lymphocyte count may result from several diseased conditions, including infection, autoimmune reactions, cancers, and cancer therapies, including radiotherapy and chemotherapy. Moreover, lymphopenia is frequently observed in patients with advanced stage cancer rather than a localized disease^23^, which influences tumor control, recurrence, and survival in various solid tumors^24^. Further, the counts of tissue infiltrating lymphocytes, which are one of the important predictive factors in immunotherapy, were found to positively correlate with ALC and negatively correlate with ANC in breast cancer^25,26^.

When considering the function and role of these blood cells in the systemic inflammation, immune system, and progression or suppression of malignancies, the prognostic importance of these cells in HCC can also be predicted. Moreover, several studies have been performed to determine the correlation between prognoses and relative proportions of each cell type, including NLR, PLR, and LMR^27–30^, which suggest a significant difference in recurrence and survival based on the proportion of each blood cell type, despite administering different treatment modalities in patients with HCC^9–11^. However, studies focusing on prognostic importance of absolute count itself of each cell type are rarely conducted, except for few studying lymphopenia and thrombocytopenia.

Therefore, our study might provide informative results, where we have analyzed the correlation between absolute counts of each blood cell type and disease prognosis and inferred their role in the microenvironment of HCC, using a relatively large homogenous patient cohort from a single tertiary institution. Here, our analysis indicates that ANC, AMC, and APC are negatively correlated with OS, whereas ALC is positively correlated with OS, most maximally at cutoff values of 3917, 488, 22,100, and 1379, respectively.

Consistent with results of studies evaluating the relationship between systemic inflammation and tumor burden in various tumors^27–30^, the elevation in ANC, AMC, and APC and decrease in ALC were significantly correlated to advanced stage of HCC. Moreover, significant association of these blood cells was observed with the Child–Pugh class and ALBI grade, which indicate the liver function status. These results suggest that each blood cell functions in tumor growth and/or suppression and exacerbation or mitigation of background hepatitis via its role in systemic inflammation or immune reaction, similar to previous reports^16,18^.

The ABC prognostic model, based on blood cells including ANC, AMC, ALC, and APC, showed an impressive prognostic ability, although less efficiently than the well-studied prognostic models viz*.* JIS or ALBI-T^31,32^. However, using groups based on the ABC model, prognosis could be clearly distinguished in the subgroups with the best prognosis identified by ALBI-T, which is one of the most reliable prognostic models in HCC. Therefore, the ABC model might be used complementary with the established staging systems in HCC, including UICC, JIS, and ALBI-T, and help to precisely predict prognosis.

However, the present study has several limitations. First, this study was conducted in Korea, an HBV endemic area, and more than three-quarters of all patients had HBV-related HCC in both training and validation sets. Therefore, it would be difficult to extrapolate the results obtained in our study with patients in the Western countries, where majority of the patients present with hepatitis from causes other than viral infections. Second, we have performed analysis using only basic complete blood counts with differential, without analyzing the specific subtypes of each blood cells with different functions. Therefore, to understand how each blood cell functions in tumor progression or suppression in HCC, a study focusing on the association of each specific blood cell subtype with clinical outcomes should be performed.

## Conclusions

The present study establishes a prognostic ABC model that analyzes the association between the absolute count of blood cells and the prognosis in patients with HCC, by providing additional prognostic distinction in the ALBI-T model representing liver function (ALBI) and the disease burden (UICC staging system). Further, these findings suggest that the absolute counts of blood cells associated with systemic inflammation and immune response can correlate with liver function and tumor burden and also independently predict prognosis.

Taken together, the findings of the present study may aid in more accurate prognosis in patients with newly diagnosed HCC and provide important clues for future research focusing on immune response and systemic inflammation in HCC.

## Materials and methods

### HCC registry

The study was performed with the HCC registry of the Samsung Medical Center (SMC), Korea. The registry was initiated from January 2005 and continued so far, and the baseline data were collected in a prospective manner from each patient previously untreated and newly diagnosed with HCC at the SMC. The diagnosis of HCC was confirmed either histologically or clinically based on the guidelines proposed by the Korean Liver Cancer Study Group. The principle of the HCC registration of the patients diagnosed with HCC is as follows: (1) patients not treated previously for HCC; (2) patients received at least one treatment or care for HCC in SMC; and, (3) patients not currently diagnosed or undergoing treatment for malignancy other than HCC.

Further, the baseline data collected in this registry at the time of HCC diagnosis were as follows: patients’ demographic data including age, sex, weight and height, date of HCC diagnosis, background liver disease, etiology, number of HCC lesions, maximum diameter of the primary tumor, vessel involvement and/or bile duct invasion, presence of extrahepatic metastasis, stage of the disease (Barcelona Clinic Liver Cancer [BCLC] and modified International Union for Cancer Control [UICC]), ECOG performance status, presence of ascites or encephalopathy, laboratory results related with liver disease or function (albumin, bilirubin, prothrombin time [PT], activated partial prothromboplastin time [aPTT], aspartate aminotransferase [AST], alanine aminotransferase [ALT], alpha-fetoprotein [AFP], the protein induced by vitamin K absence or antagonist-II [PIVKA-II], creatinine, and sodium), Child‐Pugh score/classification, and initial treatment modality for HCC. Moreover, the laboratory tests, including complete blood count with differential and electrolyte profile, were performed in all cases registered at the HCC registry at the time of diagnosis. Therefore, though these data were not recorded in the registry, the outcomes could be also obtained from the medical records. About 2 mL peripheral venous blood samples were collected in the ethylenediaminetetraacetic acid (EDTA) anticoagulant vacuum tube and analysis was performed by using a fully automated hematologic analyzer (XN-9000, Sysmex, Kobe, Japan) in our department of Laboratory Medicine and Genetics.

From this HCC registry, a patient cohort registered from January 2005 to December 2013 (training set, 6619 patients with HCC) was recruited to evaluate the prognostic significance of absolute counts of each blood cell on the OS and a cohort (validation set, 2084 patients with HCC) registered between January 2014 and December 2016 allowed validation of the findings in the training set. The baseline clinical and tumor characteristics of the training and validation sets and their comparative analysis have been presented in Table 3. The median age was 57 years (range 13–88 years) in training and 59 years (21–89 years) in validation set (*p* < 0.001). More than three quarters of all the patients were male and related with HBV infection. About 85% patients of training and 90% patients of validation set had Child–Pugh class A status (*p* < 0.001). There was no significant difference in terms of T and N stage between two sets. Additional details of the HCC registry of SMC have been described previously^33^.

**Table 3:** The baseline characteristics of patients in the training and validation sets.

Variables	Training set (*n* = 6619)	Validation set (*n* = 2084)	*p *value
**Age (years)**			
Median (range)	57 (13–88)	59 (21–89)	< 0.001
**Sex**			
Male	2875 (82.0)	2411 (77.5)	0.64
Female	633 (18.0)	699 (22.5)	
**Cause of hepatitis**			
HBV	4970 (75.1)	1554 (74.6)	0.002
HCV	640 (9.7)	182 (8.7)	
HBV/HCV	59 (0.9)	19 (0.9)	
Alcohol	287 (4.3)	134 (6.4)	
Unknown	663 (10.0)	195 (9.4)	
**ECOG performance status**			
0	6020 (91.0)	2004 (97.9)	< 0.001
1	467 (7.1)	41 (8.1)	
2	62 (0.9)	1 (0.0)	
3	46 (0.7)	0 (0.0)	
4	24 (0.4)	1 (0.0)	
**Child–Pugh Class**			
A	5602 (84.6)	1856 (89.1)	< 0.001
B	895 (13.5)	199 (9.5)	
C	122 (1.8)	29 (1.4)	
**ALBI grade**			
I	3509 (53.0)	1503 (72.1)	< 0.001
II	2787 (42.1)	537 (25.8)	
III	323 (4.9)	44 (2.1)	
**BCLC stage**			
0	1020 (15.4)	398 (19.1)	< 0.001
A	3016 (45.6)	789 (39.7)	
B	761 (11.5)	175 (8.4)	
C	1643 (24.8)	694 (33.3)	
D	179 (2.7)	28 (1.3)	
**Portal vein invasion**			
Vp0	5489 (82.9)	1476 (70.9)	< 0.001
Vp1	431 (6.5)	336 (16.1)	
Vp2	182 (2.7)	1 (0.0)	
Vp3	125 (1.9)	137 (6.6)	
Vp4	392 (5.9)	133 (6.4)	
**T stage**			
1	1218 (18.4)	366 (18.2)	0.19
2	2916 (44.1)	856 (42.5)	
3	1953 (29.5)	602 (29.9)	
4	532 (8.0)	191 (9.5)	
**N stage**			
0	6195 (93.6)	1952 (93.7)	0.88
1	424 (6.4)	131 (6.3)	
**M stage**			
0	6313 (95.4)	2026 (97.3)	< 0.001
1	306 (4.6)	57 (2.7)	
**AFP (ng/mL)**			
Median (range)	38 (1–600,000)	20 (1–200,000)	< 0.001
**PIVKA-II (mAU/mL)**			
Median (range)	53 (2–75,000)	77 (6–75,000)	< 0.001
**Primary treatment**			
Liver transplantation	130 (2.0)	21 (1.0)	< 0.001
Hepatectomy	1873 (28.3)	781 (37.5)	
Radiofrequency ablation	1321 (20.0)	350 (16.8)	
TACE	2630 (39.7)	693 (33.3)	
Systemic therapy	255 (3.9)	77 (3.7)	
Radiotherapy	32 (0.5)	37 (1.8)	
None	378 (5.7)	125 (6.0)	

*ECOG* Eastern Cooperative Oncology Group, *HBV* hepatitis B virus, *HCV* hepatitis C virus, *BCLC* Barcelona Clinic Liver Cancer, *ALBI* albumin–bilirubin, *AFP* alpha-fetoprotein, *PIVKA-II* Protein induced by vitamin K absence or antagonist-II, *TACE* trans-arterial chemo-embolization.

### General principle of HCC management in SMC

In the management of HCC during the period of the present study, appropriate treatment was provided for underlying liver disease. Moreover, the primary treatment measure for the patients was aggressive local control of primary HCC lesion and/or liver function maintenance under conditions of good liver function and performance status.

Surgical resection or radiofrequency ablation (RFA) were preferentially considered in each case where management was indicated, and the final decision was made based on the liver function/status, location of the tumor, and/or ease of administering the modality. Transarterial chemo-embolization (TACE) was used as a secondary option to control loco-regional disease in patients not suited for surgical resection or RFA. External beam radiotherapy was combined with TACE, mainly in the patients in whom TACE would be ineffective because of following reasons: maximum tumor diameter larger than 10 cm, Vp3 or Vp4 portal vein tumor invasion, or ineffective TACE history above two times. Since 2009, stereotactic body radiotherapy with or without TACE has been applied to HCC, including unresectable and small tumor less than 3 cm unsuitable for RFA. Systemic therapy, mainly sorafenib, was considered mostly in cases who were unsuited or in whom other loco-regional modalities had failed, and/or who showed multiple distant metastases.

The treatment policy was decided by the HCC tumor board participating hepatologists, surgeons, diagnostic radiologists, intervention radiologist, medical oncologists, and radiation oncologist administered once a week, if the case is judged that the application of established treatment protocol does not fit well. The details of the multidisciplinary team approach for HCC in our institution have been described previously^33^.

### Statistical analyses

The prognostic significance of the variables, including absolute blood counts, was evaluated with OS of the patients. The OS was measured as date of HCC diagnosis to that of mortality based on the medical record assessed on September 24, 2019, and/or the survival data until December 2017, from the Korean National Statistical Office, or the last follow-up visit to our institution. The cutoff value of absolute blood counts was determined using the log-rank test statistic proposed by Contal and O'Quigley^34^.

The chi-squared test or Fisher’s exact test for categorical variables and the Wilcoxon rank sum test for continuous variables were used to evaluate the correlation between blood cells as a binary variable obtained by each optimal cutoff value and other prognostic factors. The prognostic significance of each blood cell as a binary variable on OS was analyzed using the Cox proportional hazards model. For comparing the prognostic performance between the model based on blood cells and the pre-established prognostic models for HCC, a C-index and iAUC of the maximum likelihood estimate analysis were used. Further, the distribution of the C-index and iAUC values was examined using 1000 bootstrap samples with replacement from the training set cohort data. A prognostic model is considered to have a better performance if there is a higher C-index and/or iAUC value. Statistical analysis was performed using SPSS software version 24.0 for Windows (SPSS, Chicago, IL, USA), SAS version 9.4 (SAS Institute, Cary, NC), and R 3.6.2 (Vienna, Austria; http://www.R-project.org/). A *p* value < 0.05 was considered statistically significant.

### Ethics statement

The study protocol conformed to the ethical guidelines of the World Medical Association Declaration of Helsinki and was approved by our institutional review board and waived the informed consent requirements because we used only de-identified, routinely collected data during hospital visits (2020-03-158; Samsung Medical Center Institutional Review Board).

## Supplementary Information

Supplementary Figures.

Supplementary Tables.

## Abbreviations

ALC: Absolute lymphocyte count
AMC: Absolute monocyte count
ANC: Absolute neutrophil count
APC: Absolute platelet count
ECOG: Eastern Cooperative Oncology Group
HCC: Hepatocellular carcinoma
NET: Neutrophil extracellular trap
NLR: Neutrophil-to-lymphocyte ratio
LMR: Lymphocyte-to-monocyte ratio
PLR: Platelet-to-lymphocyte ratio
RFA: Radiofrequency ablation
SMC: Samsung Medical Center
TACE: Transarterial chemo-embolization
