# Supplementary material for: Clinical importance of the absolute count of neutrophils, lymphocytes, monocytes, and platelets in newly diagnosed hepatocellular carcinoma
Source: Sci Rep. 2021 Jan 28;11:2614. doi: 10.1038/s41598-021-82177-5 (PMC7844216; doi:10.1038/s41598-021-82177-5)

**Article type: Original article**

**Clinical importance of the absolute count of neutrophils, lymphocytes, monocytes, and platelets in newly diagnosed hepatocellular carcinoma**

Jeong Il Yu^1,^*, Hee Chul Park^1,2,^*, Gyu Sang Yoo^1^, Changhoon Choi^1^, Moon Seok Choi^3^, Heerim Nam^4^, Sun-Young Baek^5^, and Minsu Park^5,6^

Departments of ^1^Radiation Oncology and ^3^Medicine, Samsung Medical Center, Sungkyunkwan University School of Medicine, Seoul, Republic of Korea

^2^Department of Medical Device Management and Research, Samsung Advanced Institute for Health Sciences and Technology, Sungkyunkwan University, Seoul, Republic of Korea

^4^Department of Radiation Oncology, Gangbook Samsung Hospital, Sungkyunkwan University School of Medicine, Seoul, Republic of Korea

^5^Statistics and Data Center, Research Institute for Future Medicine, Samsung Medical Center, Seoul, Republic of Korea

^6^Department of Statistics, Keimyung University, Daegu, Republic of Korea

Jeong Il Yu and Hee Chul Park contributed equally to this work as corresponding authors.

**Co-corresponding author:** Jeong Il Yu, M.D., Ph.D.

Department of Radiation Oncology, Samsung Medical Center, Sungkyunkwan University School of Medicine, 81 Irwon-ro, Gangnam-gu, Seoul 06351, Korea

Tel.: 82-2-3410-9598; Fax: 82-2-3410-2619; E-mail: ro.yuji651@gmail.com

**Co-corresponding author:** Hee Chul Park, M.D., Ph.D.

Department of Radiation Oncology, Samsung Medical Center, Sungkyunkwan University School of Medicine, 81 Irwon-ro, Gangnam-gu, Seoul 06351, Korea

Tel.: 82-2-3410-2612; Fax: 82-2-3410-2619; E-mail: hee.ro.park@ gmail.com

**Running title:** absolute blood counts in HCC

**Supplementary Figure Legends**

Fig. 1. Kaplan-Meier overall survival (OS) curves of subgroups categorized with HBV related HCC or non-HBV related HCC based on the newer ABC model in training and validation sets: Although there were statistical significant difference in both groups and both sets, the difference in OS curves according to the ABC model was more prominent in the patients had HBV related HCC than in non-HBV related HCC patients.


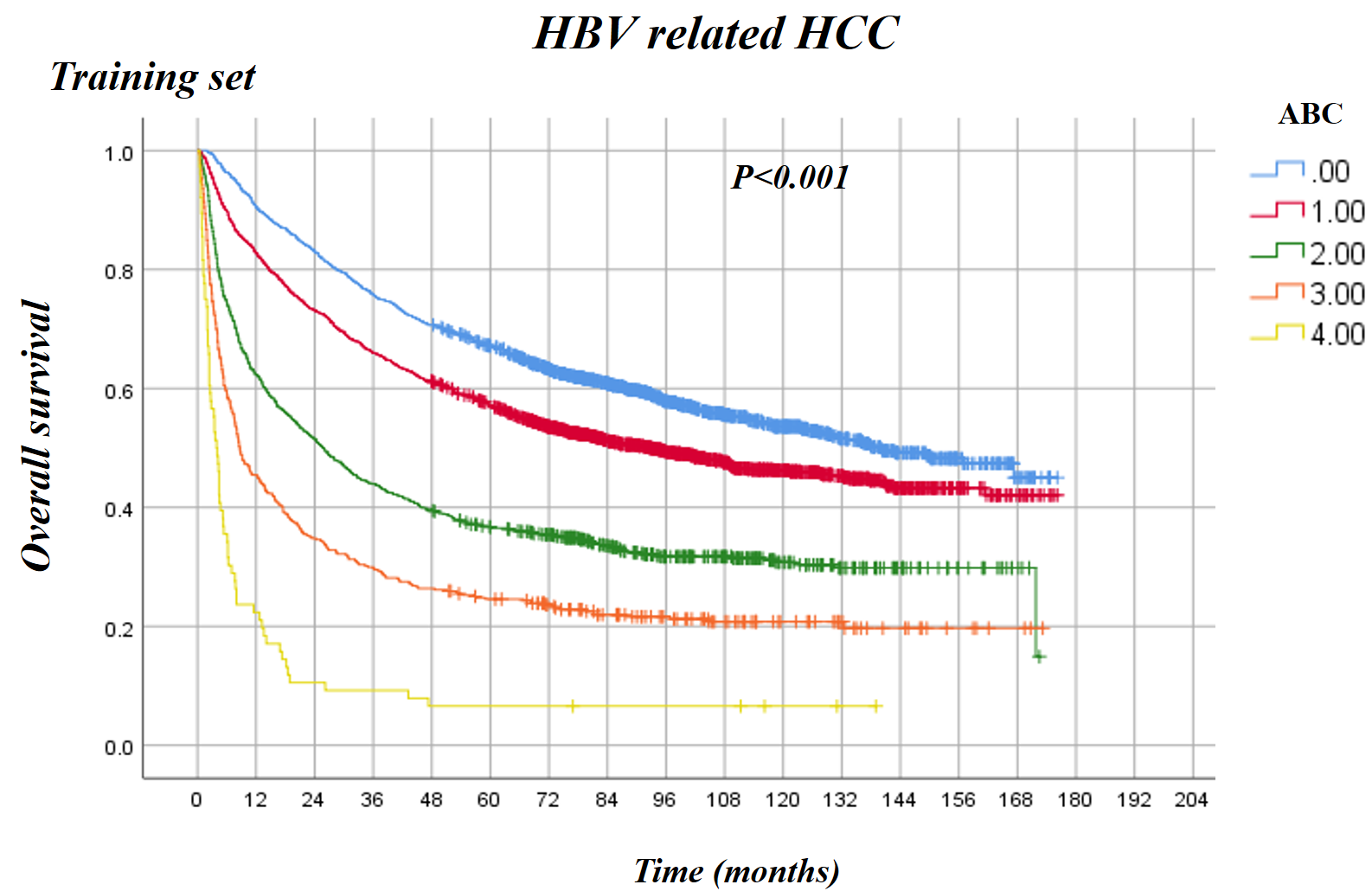

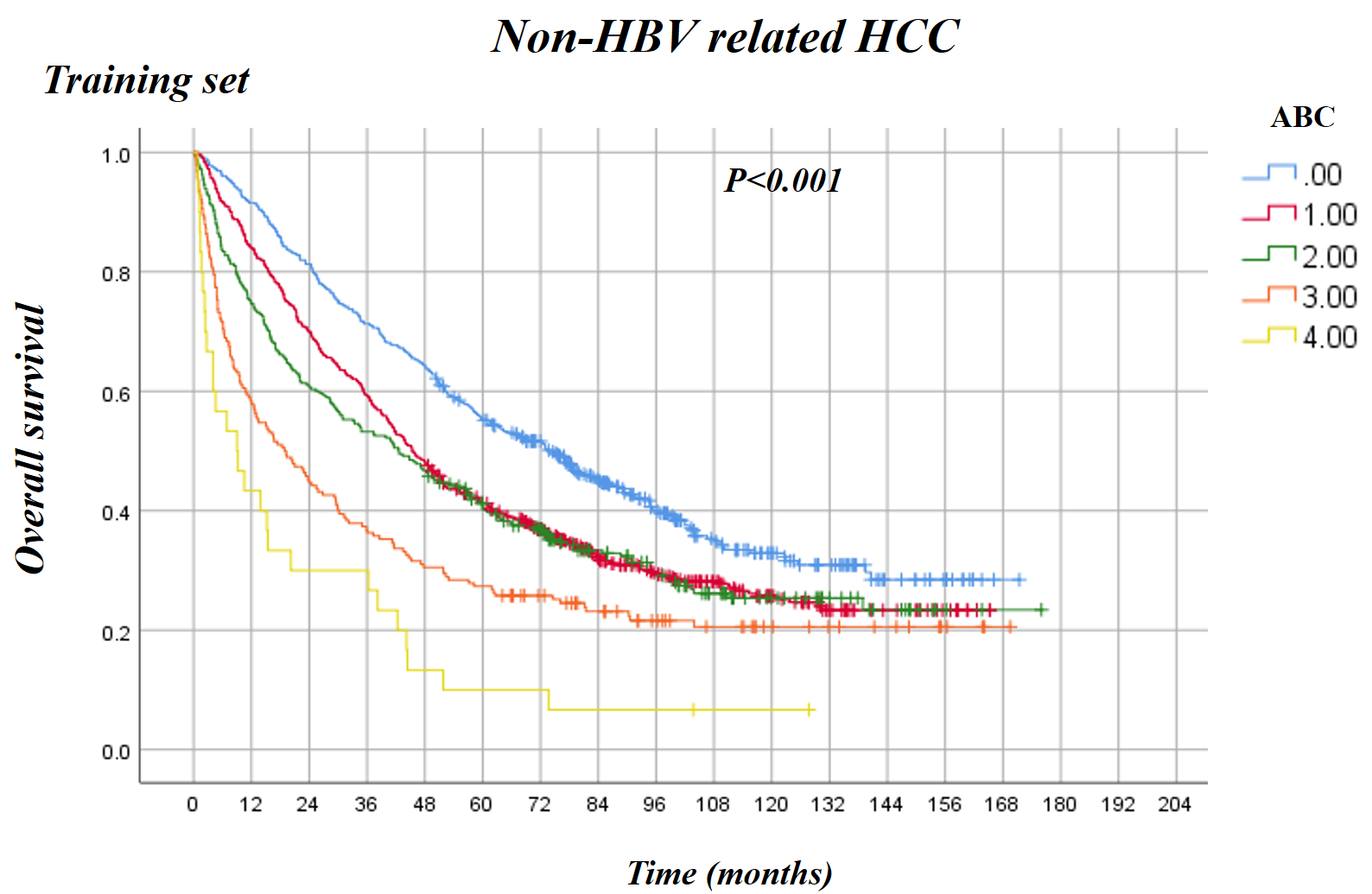


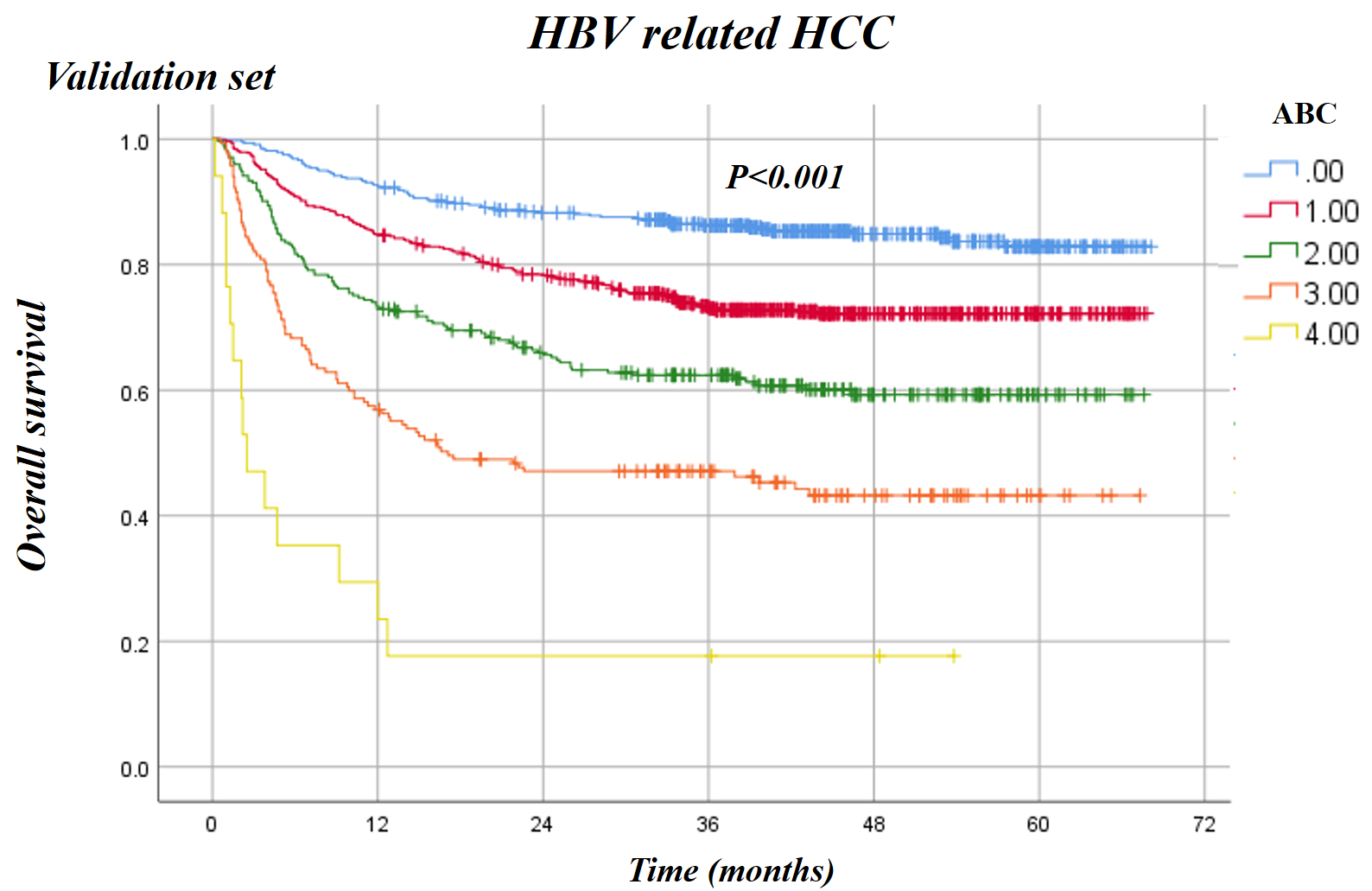

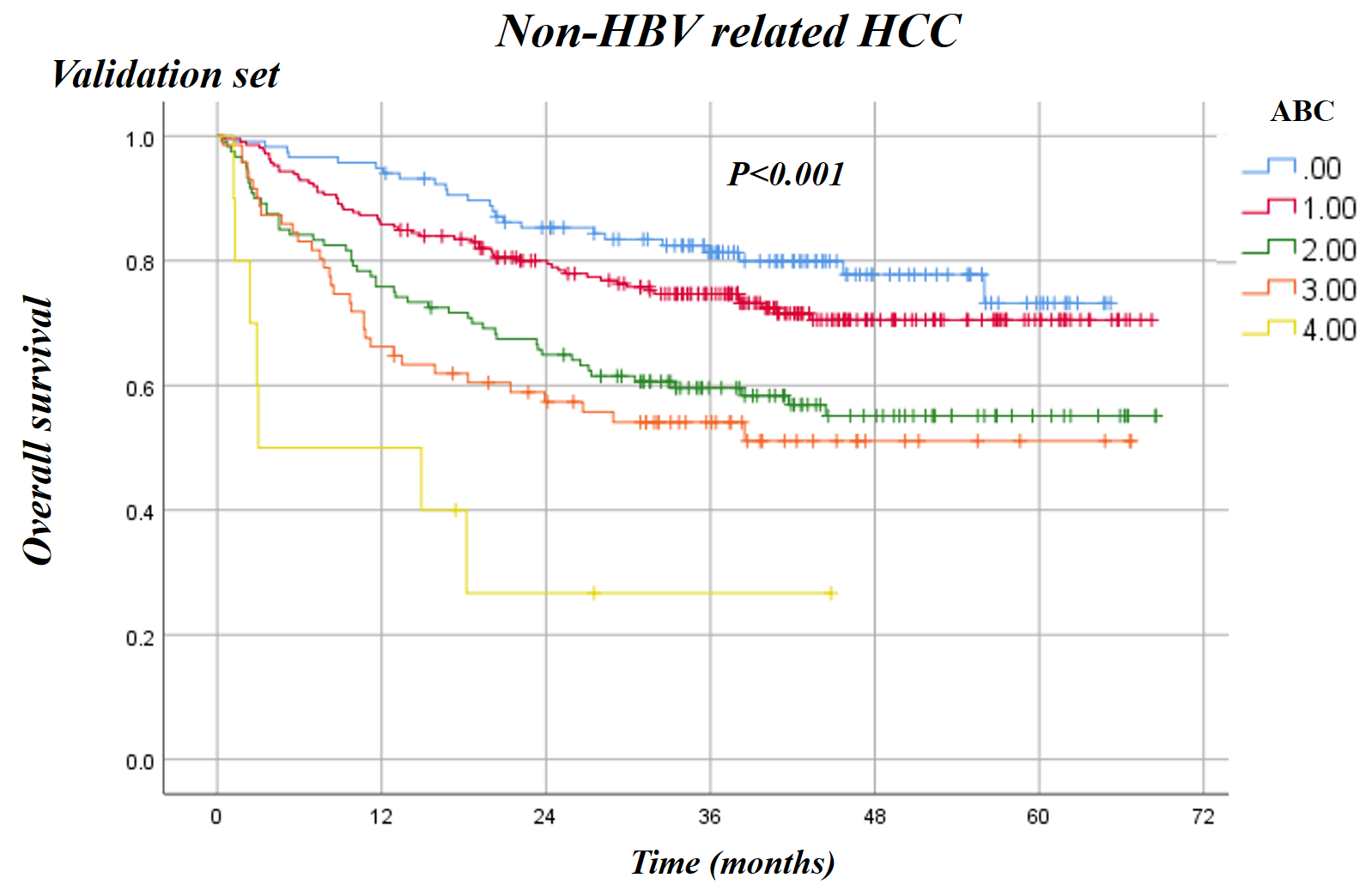


**Fig. 2.** Kaplan-Meier overall survival (OS) curves of subgroups categorized with Child-Pugh class or UICC stage based on the newer ABC model in training and validation sets: the OS based on the ABC model could be significantly distinguished regardless of the baseline liver function status (A) or tumor burden (B) in the training and validation sets.


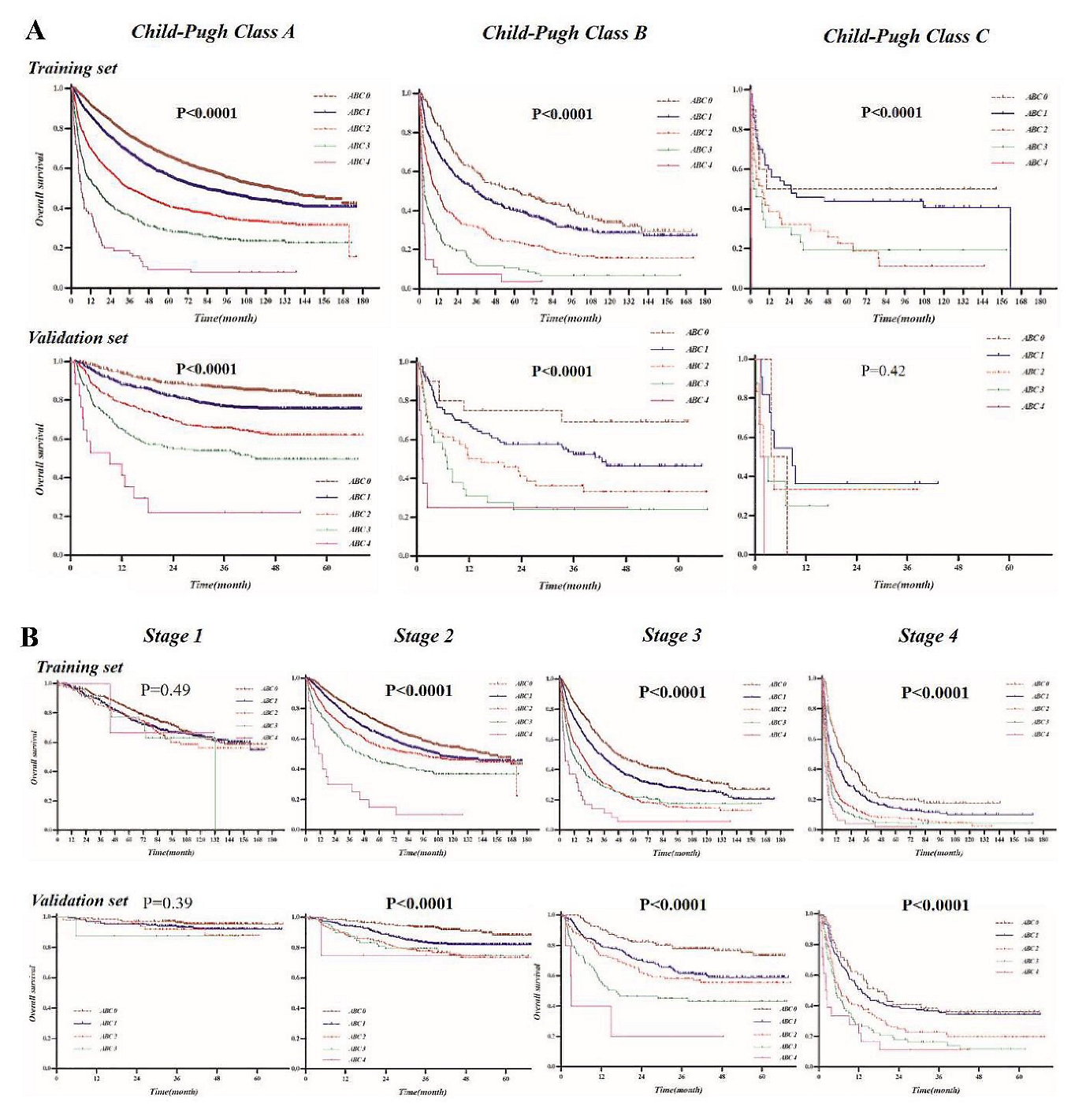


**Fig. 3.** Kaplan-Meier overall survival (OS) curves based on the albumin-bilirubin (ALBI), JIS, ALBI-T, and the newer ABC model: the ABC model showed relatively inferior performance than JIS and ALBI-T, especially in the group that showed the best survival outcome.


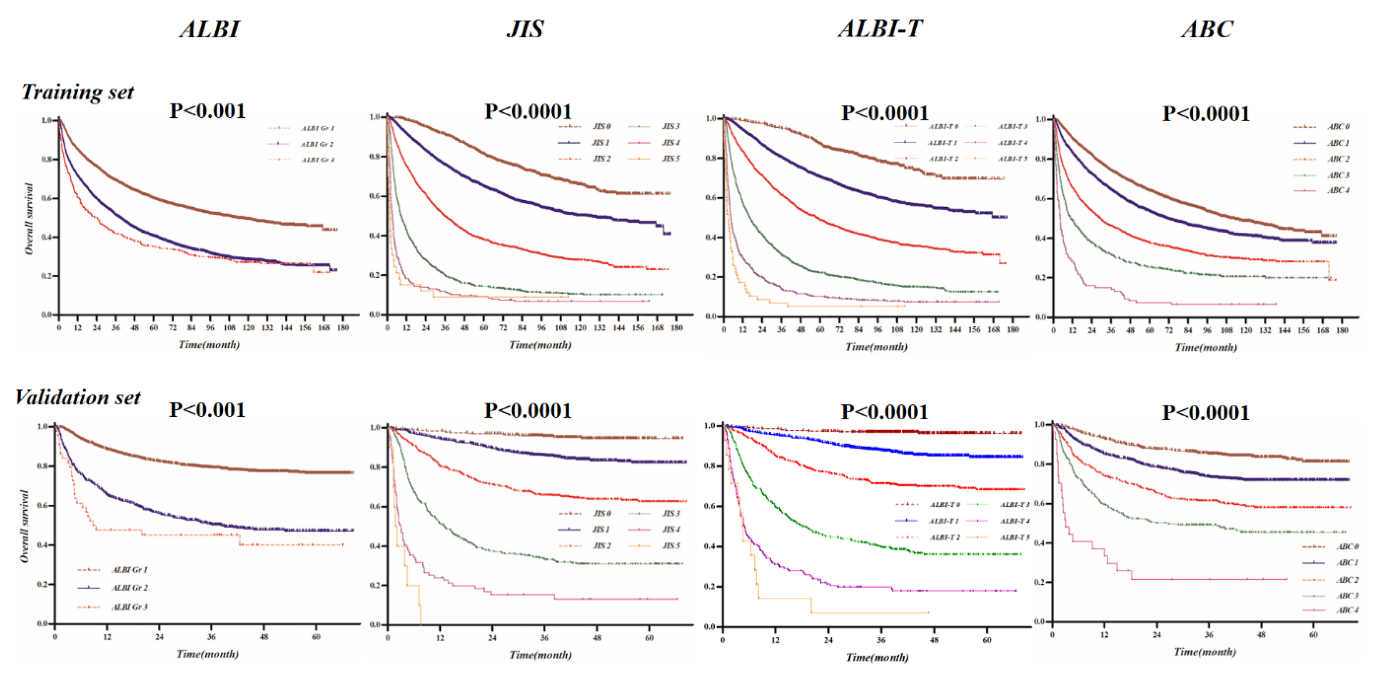


**Fig. 4.** Kaplan-Meier overall survival (OS) curves in the subgroups of albumin-bilirubin (ALBI)-T based on the ABC model: significant prognostic differences could be observed in some subgroups in the training (A) and validation (B) sets.


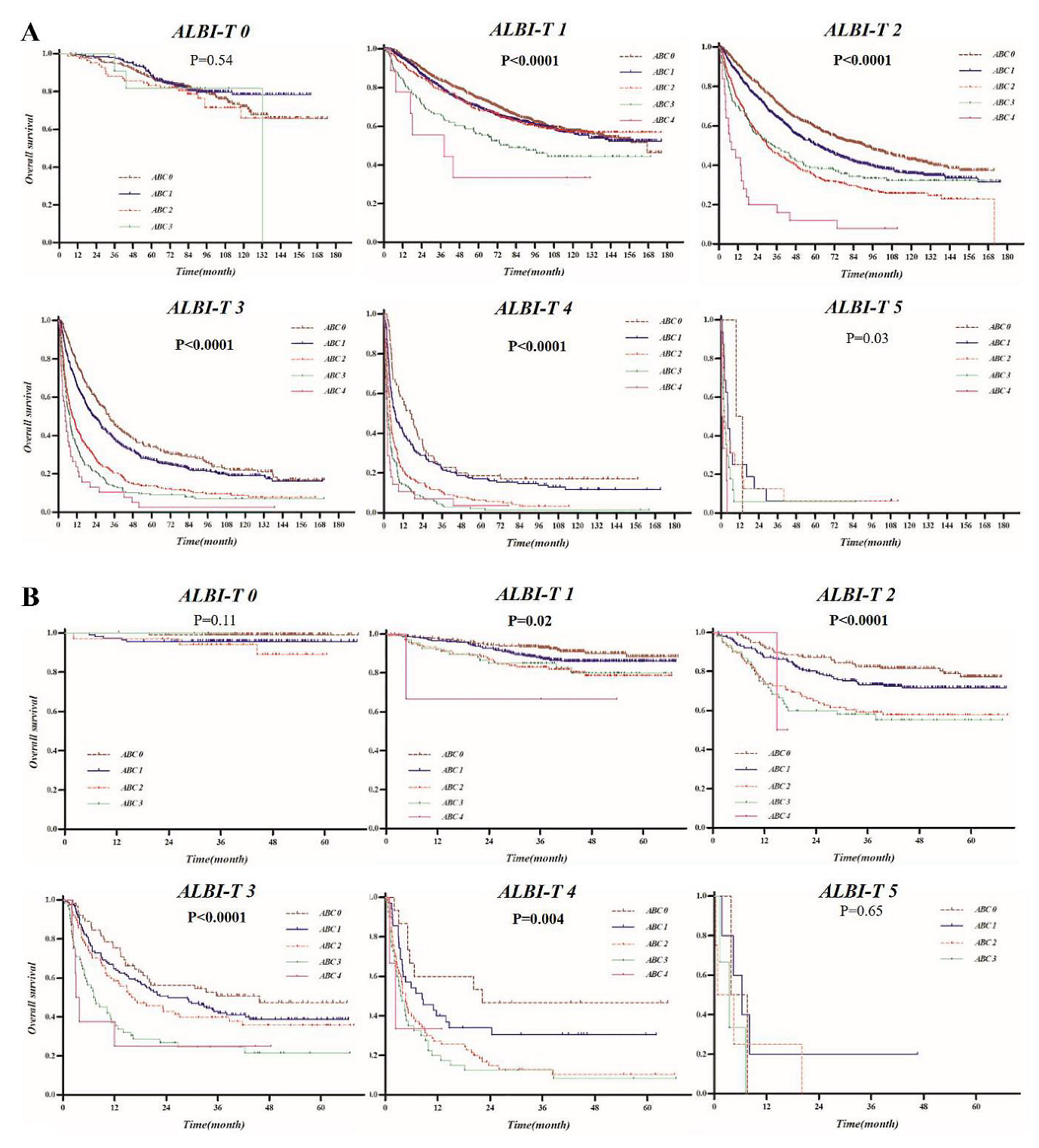

Supplement: Supplementary file 1 — Supplementary Figures. [file 41598_2021_82177_MOESM1_ESM.docx]
